# Impact of Microalgae-Bacteria Interactions on the Production of Algal Biomass and Associated Compounds

**DOI:** 10.3390/md14050100

**Published:** 2016-05-19

**Authors:** Juan Luis Fuentes, Inés Garbayo, María Cuaresma, Zaida Montero, Manuel González-del-Valle, Carlos Vílchez

**Affiliations:** 1 Algal Biotechnology Group, Ciderta and Faculty of Sciences, University of Huelva and Marine International Campus of Excellence (CEIMAR), Huelva 21007, Spain; juanlufc@gmail.com (J.L.F.); garbayo@uhu.es (I.G.); maria.cuaresma@dqcm.uhu.es (M.C.); mariazaida.montero@hotmail.com (Z.M.); 2 BioAvan S.L., Parque Empresarial Torneo, Geología 97, Sevilla 41015, Spain; manolo@bioavan.com

**Keywords:** microalgae, microalgae-bacteria interactions, microalgae production, aquaculture

## Abstract

A greater insight on the control of the interactions between microalgae and other microorganisms, particularly bacteria, should be useful for enhancing the efficiency of microalgal biomass production and associated valuable compounds. Little attention has been paid to the controlled utilization of microalgae-bacteria consortia. However, the studies of microalgal-bacterial interactions have revealed a significant impact of the mutualistic or parasitic relationships on algal growth. The algal growth, for instance, has been shown to be enhanced by growth promoting factors produced by bacteria, such as indole-3-acetic acid. Vitamin B_12_ produced by bacteria in algal cultures and bacterial siderophores are also known to be involved in promoting faster microalgal growth. More interestingly, enhancement in the intracellular levels of carbohydrates, lipids and pigments of microalgae coupled with algal growth stimulation has also been reported. In this sense, massive algal production might occur in the presence of bacteria, and microalgae-bacteria interactions can be beneficial to the massive production of microalgae and algal products. This manuscript reviews the recent knowledge on the impact of the microalgae-bacteria interactions on the production of microalgae and accumulation of valuable compounds, with an emphasis on algal species having application in aquaculture.

## 1. Introduction

The evolution of algae and bacteria cannot be understood properly if taken individually. They influence ecosystems together and represent all conceivable modes of mutual interactions between different organisms, ranging from mutualism to parasitism. Algae and bacteria synergistically affect each other´s physiology and metabolism, although bacteria have often been considered as mere contamination of algae cultures. However, in the last few years, the scenario has changed. Nowadays, algae-bacteria interactions are being seen as promising in biotechnology, as some recent studies have shown a positive effect of algae-bacteria interaction on algal growth and flocculation processes, which are the essential steps in algal biotechnology [1,2,3,4,5,6]. Consequently, the knowledge and control of the mechanisms involved in microalgae-bacteria interaction could help improve the algal biomass in microalgae production processes. This manuscript reviews the recent knowledge on the microalgae-bacteria interaction and how it influences the production of microalgae and associated algal compounds. The relevance of microalgae-bacteria interactions in improving the microalgal role in aquaculture is also reviewed.

## 2. Algae-Bacteria Interactions: Relevance and Types

The plankton communities, in which many microalgae and bacteria are included, influence the global carbon cycle and therefore the climate. It was demonstrated that heterotrophic bacteria not only decompose organic matter but also promote plant growth by certain complex communication mechanisms and nutrient exchange [7]. In last few years, the same phenomenon has also been observed for algae in the context of coevolution [3,5,8,9]. Therefore, any innovation in algal biotechnology should always take into account the existing relationship between algae and bacteria. They influence the ecosystem together in a natural way, and therefore could potentially be implied in future biotechnology industry [10,11].

Algae-bacteria interactions cover all possible forms of symbiotic relationships, though, many types of interactions in the planktonic zone have not yet been completely explored, mainly due to the onerous task of separating the partners, which are naturally bound to each other [12]. For example, the interaction between *Emiliania huxleyi*—a single cell marine microalga—and *Roseobacter* has most widely been studied. These mutual interactions are extremely species specific as the microenvironment of each alga is different. In the examples of microalgae-bacteria interactions studied thus far, nutrient exchange seems to play a major role. Micronutrients like vitamins [13,14] and macronutrients like nitrogen and carbon [3,14,15,16] are usually exchanged between algae and bacteria. In addition, plant hormones excreted from bacteria also promote algal growth [14]. Both algae and bacteria alter their metabolism to meet each other’s needs. Some studies suggest that such inter-regulation plays a major role in these interactions, as in the case of the *Roseobacter* [14].

### 2.1. Mutualism

Mutualism is a biologic interaction in which two or more partners of different species benefit each other [6,8,12]. A typical example of mutualism is that a bacterial species supplies vitamin B_12_ to an algal partner in exchange for fixed carbon [13,17]. However, mutualism is not only limited to micronutrient supply from bacteria [18], as there are studies highlighting the role of *Azospirillum*, *Mesorhizobium* and *Rhizobium* sp. in algal growth promotion and *vice versa* [2,3,19,20], and even as nitrogen suppliers in oligotrophic environments. Cho *et al.* demonstrated that when some algae are grown with an artificial consortium of mutualistic bacteria, they supply fixed organic carbon to the consortium, and in return, they show enhanced growth [21]. Consequently, it was hypothesized that the bacterial consortium might be providing organic and/or inorganic compounds that can be metabolized by the alga, thus promoting algal growth. Such exchanges between biotic communities in aquatic ecosystems have a huge role in cycling of nitrogen, sulphur, carbon and phosphorus [22,23,24,25,26,27,28].

### 2.2. Commensalism

Commensalism is a relationship in which only one partner benefits. Commensals could be considered as non-interacting partners [29]. Microorganisms that belong to the phycosphere represent a bacterial diversity dwelling on the algal surface [21]. However, there is a very faint line that separates mutualism and commensalism, and even parasitism, and environmental factors may shift an interaction from one type to another. In this sense, there are studies that partially demonstrate the role of nutrient availability, N:P ratio and light intensity in the shift from mutualism to parasitism and *vice versa* via commensalism, although the mechanisms behind such shifts still remain unclear [12,30]. The interaction between *Chlamydomonas reinhardtii* and heterotrophic bacteria is another example of commensalism. *C. reinhardtii* uses vitamin B_12_ delivered by heterotrophic bacteria, although the bacteria do not make use of the organic carbon released by the alga [16].

### 2.3. Parasitism

Parasitism is a well-studied interaction in which one species benefits at the expense of the other and exerts negative effects on it. Normally, the parasite is smaller in size and needs the host to be alive. Many bacteria are known to negatively affect algae, and, therefore, they have been proposed as microalgae and cyanobacterial bloom controlling microorganisms [31,32,33]. However, sometimes algae are also parasitic [34], for instance, about 10% of known red algae are parasitic [35]. In the case of bacterial parasitism on algae, the algal cell is lysed by the action of glucosidases, chitinases, cellulases and other enzymes [33,36]. Once the algal cell is lysed, the bacteria can use intracellular algal compounds as nutrients. However, there is one more form of parasitism, where a competition for existing nutrients occurs that results in slower growth rates of algae [12]. An apparent or incidental altruism has recently been reported, in which an individual acts for the exclusive benefit of another; and it can be self-driven or driven by the beneficiary [37]. We speculate that such an altruistic relationship might also be taking place in some of the bacterial-microalgal consortia. In general, most of these associations usually occur in close proximities. For example, the parasitic bacteria are usually present near the algal cell wall to facilitate its degradation [33] and the habitats play an important role in the ecophysiology of these organisms. Parasites in general have wide-ranging applications in industrial biotechnology. For example, microbial cellulases, hemicelluloses and pectinases, obtained from such parasites, are currently being used in food, brewery, wine, textile and paper industries, among others [38]. Chitinolytic enzymes are also used for the preparation of pharmaceutical oligosaccharides and for control of pathogenic microorganism transmission [39].

In summary, a variety of interactions between algae and bacteria have been described, which can range from beneficial to detrimental to algal growth. The control of some of these interactions may serve as a highly useful tool to either stimulate the production of a given microalgal species, to control algal blooms, or to even harvest algal biomass at a low cost.

## 3. Algae-Bacteria Interactions: Effects on Biomass Production

**Figure 1:**
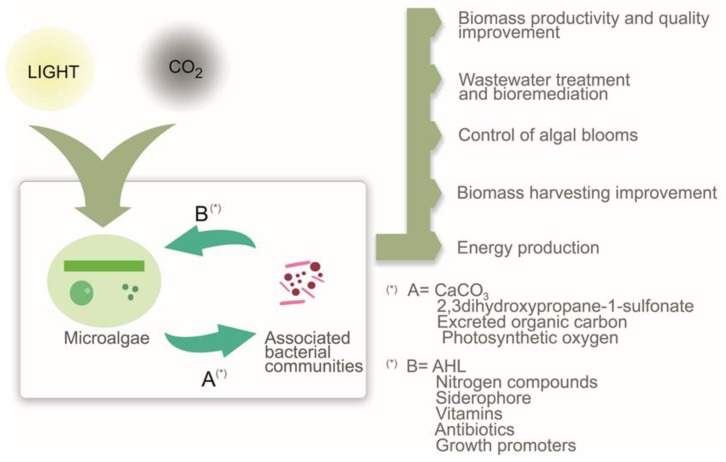
Interaction between microalgae and bacteria. Some of the main chemical mediators (**A,B**) and potential applications. Micronutrients like vitamins and macronutrients like nitrogen, oxygen and carbon usually exchange between algae and bacteria (Improvement in biomass productivity and quality). Photosynthetic oxygen can be consumed by bacteria creating a suitable environment for algal hydrogen production (energy production). A typical example of mutualism is that the bacteria supply vitamin B_12_ (depicted by molecule B in the Figure) to the algae in exchange for fixed carbon (depicted by molecule A in the Figure). Antibiotics can be produced by the bacteria for algal protection against other microorganisms (mutualism/commensalism) or for algal lysis (parasitism, control of algal blooms). AHL (acyl-homoserine lactone) is produced by bacteria and is involved in biofilm formation between bacteria and algae cells (wastewater treatment and biomass harvesting improvement).

Traditionally, efforts have been paid to obtain axenic algal monocultures for developing biomass production processes. However, it is nowadays recognized that the interactions between microalgae and microorganisms have potential, with special applicability in aquaculture, to improve algal biomass production and to enrich this biomass with compounds of industrial interest such as lipids and carbohydrates. In this respect, the general bacterial attributes that may be of interest in the interaction with microalgae, and which might affect their growth, include motility, chemotaxis, type IV secretion systems, *quorum sensing* systems and synthesis of growth promoters [40]. Figure 1 summarizes some of the main chemical mediators so far reported, which regulate the interactions between microalgae and bacteria and some of the potential applications derived from such interactions.

Bacterial communities associated with microalgal cultures can be very useful for their growth, for instance, they can provide vitamins for better microalgal growth which could result in low production cost of microalgal biomass and therefore in greater production efficiency. In fact, many microalgae are auxotrophic for certain vitamins, as already shown by Croft *et al.* in 2005, who found that 171 algal species among 326 species studied require an external supply of vitamin B_12_ [13]. Vitamin B_12_ is required for a proper functionality of an isoform of methionine synthase enzyme of microalgae. Indeed, it has also been estimated that approximately 25% of the existing microalgae might be auxotrophic for vitamin B_1_, and approximately 8% for vitamin B_7_ [40].

In the interactions between microalgae and bacteria, there might also be benefit to the heterotrophic organism as a result of a mutualistic relationship, as briefly explained above. Kazamia *et al.* produced co-cultures of the vitamin B_12_-dependent microalga *Lobomonas rostrata* and the bacterium *Rhizobium loti* [16]. These organisms were grown together in a culture medium which initially had neither vitamin B_12_ nor an organic carbon source, so that neither algal nor bacterial growth was possible. The obtained results showed enhanced algal growth with a mutual growth regulation between the microalga and the bacterium, and both microorganisms reached a balance in population density. This is a typical case of mutualism between bacteria and microalgae, through which the bacterium provides the microalga with vitamin B_12_ and the microalga in turn provides the bacterium with organic carbon compounds.

These mutualistic interactions described naturally occur in sea environments. Assuming that half of the carbon fixed by phytoplankton in the sea is metabolized by bacteria, Durham *et al.* made a model of the algae-bacteria interaction that occurs in the oceans. For that purpose, they used the roseobacterium *Ruegeria pomeroyi* DSS-3 and the diatom *Thalassiosira pseudonana* CCMP1335 [41]. In this specific microalga-bacterium interaction, *R. pomeroyi* supplies vitamin B_12_ to *T. pseudonana* which, in turn, excretes 2,3 dihydroxypropane-1-sulphonate into the medium, used in the bacterial metabolism as carbon source. Interestingly, Durham *et al.* observed differences in the gene expression of the diatom when grown in the presence or absence of the roseobacterium, suggesting an influence of *R. pomeroyi* DSS-3 on the metabolism of *T. pseudonana* CCMP1335 [41]. As expected, the algal growth was found to be slower without any source of vitamin B_12_ due to the auxotrophic nature of *T. pseudonana* CCMP1335 for vitamin B_12_. However, the algal growth in co-cultures with *R. pomeroyi* DSS-3 was found to be similar to that in the absence of *R. pomeroyi* DSS-3 but supplemented with vitamin B_12_. This suggests that vitamin B_12_, produced by *R. pomeroyi*, might have a positive effect on the growth of *T. pseudonana* CCMP1335, in coherence with the above discussed mutualistic algae-bacteria interactions.

The bacterial communities associated with microalgae cultures can also regenerate or fix inorganic nutrients that otherwise would not be bioavailable to them. The importance of the micronutrient iron on the growth and species composition of algal communities in the oceans is well documented [22,42]. The bioaccesibility of iron for many species of microalgae involved in the formation of blooms, including dinoflagellates and coccolithophores, depends on their close interaction with some species of bacteria [13,43,44,45,46]. As an example, Amin *et al.* reported that the microalga *Scrippsiella trochoidea* makes use of bacterial siderophores produced in its environment [22]. The most significant bacteria within this community are phylogenetically related to α-proteobacteria *Roseobacter* and γ-proteobacteria *Marinobacter*. These bacteria produce the siderophore vibrioferrin, which binds Fe (III), making it available for microalgae and the bacteria. Subsequently, microalgae use this iron in the photosynthetic processes of inorganic carbon fixation. Some part of such fixed carbon is released back to the medium as organic molecules (dissolved organic matter, DOM), so that it can be used for bacterial growth, therefore sustaining the production of siderophores. Thus, this represents an example of the nutritional feedback between photosynthetic eukaryotes and prokaryotes, which sustains the microalgae-bacteria equilibrium in microbial ocean communities.

Nitrogen has also been widely described to be one of the nutrients involved in the nutritional traffic between microalgae and bacteria in microbial communities. Nitrogen fixation under aerobic conditions by nitrogen-fixing bacteria might result in the supply of inorganic nitrogen to microalgae for growth. This has been demonstrated in co-cultures of microalgae with *Azotobacter vinelandii* [47]. Moreover, the microalgal species *Neochloris oleoabundans* and *Scenedesmus* sp. BA032 were able to use *A. vinelandii* siderophores as nitrogen source. This interaction between algae and bacteria, which can be classified as *commensalism*, could be applied in mass culture of the microalgae, for instance, to reduce the costs of nitrogen source [48]. In this context, a noticeable example, which might be considered as *mutualism*, was reported by Foster *et al.* [49], showing that cyanobacteria *Richelia intracellularis* and *Calothrix rhizosoleniae* both increase nitrogen fixation rates and growth rates in the presence of diatoms. Simultaneously, the symbiont diatoms make use of the inorganic nitrogen previously assimilated by the cyanobacteria.

The interaction between algae and bacteria also occurs in dark and under heterotrophic conditions with different results than those in the phototrophic conditions. Cell immobilization within a polymeric matrix is a suitable system to study the relationships between algae and bacteria that grow in association like a microbial community. Under heterotrophic conditions, *Chlorella vulgaris* showed an increase in the accumulation of fatty acids and total lipids when co-immobilized with *Azospirillum brasilense*, which is a growth promoter bacterium in higher plants [50]. Moreover, the co-immobilization facilitated by an external supply of d-glucose and Na-acetate as carbon sources resulted in a significant increase in the starch and carbohydrates content of *C. vulgaris* compared to the immobilized algal cells alone [51]. A similar enhancing effect on starch and carbohydrates accumulation was also found in *C. vulgaris* cells when co-immobilized with *A. brasilense* and cultivated under photoautotrophic conditions, compared to *C. vulgaris* cells immobilized alone and grown under photoautotrophic conditions [52]. All these examples demonstrate a bacterial role in the enhancement of carbon storage in microalgae, which is particularly useful in algal production under heterotrophy. Table 1 provides the examples of microalgae-bacteria interactions having positive effects on algal growth and accumulation of valuable compounds.

In microalgae-bacteria communities involved in upwelling processes, this relationship may even be more complex, changing from mutualism to parasitism according to the physiological circumstances of the microalga [53]. This concept was demonstrated with the microalga *Emiliana huxleyi*. The study showed that mutualism occurs if *E. huxleyi* cells are physiologically healthy. In this situation the microalga fixes CO_2_ by photosynthesis and synthesises methionine, cysteine and dimethylsulphoniopropionate (DMSP) [54,55]. DMSP is trapped by roseobacteria such as *Phaeobacter gallaeciensis*, which in turn produces growth promoting factors for the microalga and antibiotics that eliminate potential pathogenic bacteria for the algal cells. Under suboptimal conditions, *E. huxleyi* produces p-coumaric acid which triggers the synthesis of an algicide roseobacticide by *P. gallaeciensis*. This algicide can lyse the microalga at nanomolar concentrations. Thus, the bacterial cells get food supply from the lysed algal biomass [53].

The bacteria associated with microalgae play a key role not only in the growth but also in the composition of the microalgal biomass. The chemical composition of the microalgal biomass is certainly a key factor in aquaculture; therefore, microalgae-bacteria interactions deserve great importance in aquaculture activities. In fish farming, for instance, the composition of the food supplied at the larval stage, especially the composition in terms of fatty acids and other lipid components, will definitely determine the end nutritional quality of the produced fish [57].

The symbiotic cultures of microalgae and bacteria often result in complete elimination of contaminating bacteria in aquaculture systems. This can be explained by the principle of competitive exclusion, which is usually prominent in such ecological communities [58]. This principle, also known as Gause’s law, states that two species cannot co-exist within the same ecological niche, within the same habitat at the same time. An illustrating example of this is the mutualistic relationship between *Emiliania huxleyi* and *Phaeobacter gallaeciensis*, wherein the bacterium produces antibiotic molecules to protect the host from other bacterial pathogens [53]. Based on this principle, it is possible that, in aquaculture systems, the microalgae-associated bacteria prevent proliferation of other microorganisms potentially pathogenic to fish larvae, by producing, for example, antimicrobials. As an example, the cultures of *Tetraselmis* spp. were found to display powerful antimicrobial activities against aquaculture pathogens [59]. This feature could partly explain the high survival rate described in cultured fish larvae when the so-called “*green water*” technique is used [57,60]. In this aquaculture technique, the microalgae that are part of the marine fish larvae diet are directly grown in the tanks for fish larval production. This technique has multiple advantages, including higher quality and higher survival rate of larvae, which could also be explained by the principle of competitive exclusion.

Besides positive effects on algal growth, detrimental effects of bacteria on the biomass yield of microalgae cultures are expected in some cases. Non-axenic batch cultures of microalgae with low or even no microbial control might result in lower algal cell density, compared to pure algal cultures. In high cell density cultures, the presence of bacteria might be expected to reduce light availability to the microalgal cells. As a consequence, the yield of algal biomass in non-axenic photoautotrophic cultures might decrease. The accumulation of target value molecules, including carotenoids and fatty acids, may require supply of higher light irradiance [61]. If light availability is reduced due to the presence of significant bacterial cell density, one would expect lower yields of light-dependent accumulation of high value algal products (g per g dry weight). Moreover, in such a case, the purification of accumulated target molecules might become more expensive and technically complicated due to the lower concentration of the specific product in the cells extracts. Therefore, control of algae-bacteria interactions is indispensable in avoiding the decreased yields of algal biomass and algal-derived products.

## 4. Algae-Bacteria Interactions: Current and Promising Applications

### 4.1. Harvesting

The harvest of microalgae biomass from liquid cultures is an expensive process and may take up to one-fourth of the total biomass production cost [62]. The most widely utilized harvesting techniques are ultrafiltration with membranes [63], electrocoagulation and centrifugation, which are not yet economically feasible at large scale [64]. Other harvesting techniques including flocculation, bioflocculation and the use of magnetic-particle-based flocculants have also been explored [62,65,66]. In particular, the use of chemical flocculants can also be an efficient alternative to conventional harvesting techniques or even in combination with them [62]. However, different harvesting strategies need to be compared with the help of a quantitative cost-benefit analysis in order to propose suitable low-cost biomass production processes for any microalgal species.

Bioflocculation might also be a potentially efficient algae harvesting method; however, further studies have to be done in this field before it could emerge as a viable alternative. It has been reported that bioflocculation might be promoted by any of the specific bacterial species associated to the cultivation of specific microalgae [65]. The cultivation conditions that enhance growth of bioflocculation-promoting bacteria would consequently stimulate bioflocculation.

Microalgal aggregation by bacteria can be triggered by large polysaccharides or proteins produced by bacteria, but such aggregation has also been reported to occur by direct attachment between bacteria and microalgae. In the latter case, ionic interactions are described as the dominating mechanism of interaction [67]. The surface of algal cells contains ionisable functional groups [68] that can get deprotonated or protonated based on the pH and therefore can create surface charge. The flocculation of microalgal cells can be enhanced by electrostatic interactions developed by variation of such surface charge. For example, the aggregation of both *Nannochloropsis oceanica* and *Bacillus* sp. strain RP1137 is dependent on pH and divalent cations, and has been described to occur via neutralization of charge with calcium ions at the cell wall surface of both algae and bacteria [69].

The accurate knowledge of cell-to-cell interaction mechanisms is important to develop energetically and economically feasible methods of algal biomass harvesting.

### 4.2. Cell Disruption

With the increasing interest in using microalgal biomass for producing high-value compounds, cost-effective disruption methods need to be developed in order to avoid high production costs of algal biomass and valuable products [70]. With an external addition of enzyme cocktails, the efficiency in terms of cell disruption is competitive but causes degradation of intracellular material [71]. Induced autolysis of microalgae has been proposed as a promising cell disruption method to avoid the problems associated with the use of enzymes [67]. In some cases, for example, in the production of biohydrogen and biomethane, a pretreatment of microalgal cells can be done to make the intracellular content accessible to degrading bacteria [70]. To induce cell lysis, microalgal degradation by algicidal microorganisms and their algicidal molecules have been employed. This is a process by which bacteria, cyanobacteria, viruses and algae mainly act through attacking and killing the targeted microalgae by releasing extracellular compounds [72,73]. It is also documented that bacteria can control bloom processes by growth inhibition of diatoms and other phytoplankton members or by active lysis of algal cells [58]. To control devastating algal blooms, the inhibition of algal growth by bacteria either requires direct cell contact or can be mediated by excreted extracellular substances [74].

Information about mechanisms of interaction and communication between algal cells is still limited, but, in contrast, some information about bacteria-algae communication is available [75,76,77]. The initial step of bacteria-algae interaction is the detection of algae by quorum sensing (QS) [78,79,80,81]. Algicidal microorganisms can induce inhibition of algal growth, and even algal death and cell lysis [72]. In this respect, it is important to mention that there is a lack of knowledge on what specific molecules are produced by the bacteria interacting with algal metabolism, and what are the specific algal metabolic steps involved in such molecular interaction processes. A number of algicidal molecules that are produced by bacteria have been identified including derivatives of quinolones, pyrroles, alkaloids and enzymes. In addition, cyanobacteria and microalgae have also been found to produce algicidal molecules such as cyclic peptides, fatty acids and lipids derivatives [72,73].

Considering the available literature, the effects resulting from algicidal compounds on a microalgal cell include morphological changes, pore formation in the plasma membrane and loss of cell ultrastructure organization, formation of radical oxygen species, loss of functionality of the antioxidant systems and inhibition of photosynthesis [72,73]. The degradation of microalgae using algicidal microorganisms in co-cultures demonstrated its effectiveness as an advantageous disruption method and could facilitate and improve macromolecule recovery, for instance lipid extraction or increasing carbohydrate availability for fermentation. In addition, it can also favor organic matter degradation for biogas production [82,83].

The relevance of the cell-to-cell communication, at both intra-and inter-species level, has to be taken into account to understand the algal cell lysis process. Indeed, the detailed description of the algal cell disruption process still requires deeper knowledge of the mechanisms behind the bacteria-mediated cell lysis. Factors that might affect cell lysis efficiency include algicidal microorganism density, algicidal compound’s concentration and the sensitivity of the algae towards the algicide.

### 4.3. Energy Production

Not much is currently known about H_2_ production by algal-bacterial systems. It is well known that hydrogen production by microalgae depends on a hydrogenase enzyme activity that is highly sensitive to oxygen. Thus, stringent anaerobic conditions are required for an efficient production of hydrogen by microalgae [84]. It has been reported that such anaerobic environment suitable for algal hydrogen production might be conferred by the bacteria which can consume the O_2_ generated photosynthetically by the algae, without damaging the photosynthetic apparatus. With the help of the bacteria that consume the O_2_ evolved, the algae can capture light energy and produce H_2_ at the same time without further manipulation of the system, such as sulphur deprivation [85].

Other approach of harnessing energy from mixed cultures of photosynthetic microorganisms is the possibility of providing power from photosynthesis to a reactor [86]. Some studies assessed the performance of phototrophic microbial fuel cells to convert solar energy to electricity by coupling photosynthesis. Interestingly, these studies show that the ability of microalgae to utilize light over a wide range of wavelengths and intensities may result in a more cost effective performance of microbial fuel cells compared to conventional photovoltaic systems [87]. However, this potential advantage still has to be demonstrated. Some efforts have been exerted by some companies to commercialize these technologies [88,89].

### 4.4. Nutrient Removal and Wastewater Treatment

Algal–bacterial systems have been extensively used in the treatment of nutrient rich wastewater since the 1950s [12]. In oxidation ponds, algal-bacterial symbiosis results in sewage treatment with exchange of O_2_ and CO_2_, and NH_4_^+^ ions. However, these systems were neither aerated nor mixed; therefore, the treatment efficiency achieved was low compared to current yields. The current high rate algal ponds (HRAP) not only offer high efficiency of the process but also enhance the possibility for sewage treatment and biofuel production with a reliable yield [12].

Besides the nutrients, several toxic metal ions can also be removed by microalgae, achieving the polishing effects of a tertiary treatment. This system can be used to treat different agro-industrial wastewater [90,91]. Another study demonstrated the effective use of methane oxidizing bacteria and microalgae to eliminate methane from anaerobically treated wastewater, which is otherwise released into the atmosphere [92]. However, the biodegradation of methanol or methane in closed algal-bacterial photobioreactors requires the addition of external oxygen or inorganic carbon, a process that requires further optimization [93]. Considering the efficiency of the treatment, low energy consumption, the absence of synthetic chemicals and produced microalgae biomass, which could be further valorized, algal–bacterial sewage treatment process could be a major alternative technology to aeration based technologies like activated sludge treatment (AST) [94]. A new approach is the use of systems based on the formation of algal-bacterial biofilms. The ease of cultivation, the relatively less self-shading compared to suspended systems, and the ease of harvest are the major factors that make them popular. However, an algal-bacterial biofilm based sewage treatment system is still far from being applied at a large scale [95,96,97]. One of the constraints in the use of algal-bacterial biofilm systems is that light availability remains limited to the photic zone, just a few hundred millimeters below the water surface [98]; this therefore requires large surface systems. Some engineering based studies are required to overcome this constraint, for example, the development of the moving bed biofilm reactor that uses plastic biofilm carriers to maximize the active biofilm surface area in the reactors [99].

### 4.5. Bioremediation

The positive effect of algae-bacteria consortium for metal bioremediation has been documented [100]. Algae require several metals in small quantities for normal growth and metabolism but higher levels of the same metal are toxic. In this sense, algal-bacterial community in mutualistic interactions can detoxify and assimilate metals from metal rich environments. This process includes physical adsorption, covalent bonding, ion exchange and chemisorption, surface precipitation, redox reactions or crystallization on the cell surface. On a lesser scale, metals can be removed from the environment by active uptake into the cell to be used in their metabolism or as a defensive tool to avoid poisoning [101,102]. In spite of this fact, it would be relevant to mention that many microalgae are sensitive to low concentrations of metal ions, particularly of those not required for growth [103] and bacteria are not expected to possibly reduce such sensitivity.

The degradation of organic pollutants has also been reported, including black oil, acetonitrile, phenol, naphthalene, thiocyanate and benzopyrene and azo compounds among others [101,102,104,105]. Besides this, the degradation of toxic pesticides as monocrotophos, quinalphos, methyl parathion, DDT, atrazine and α-endosulphan was also demonstrated [10,102].

### 4.6. Sustainable Aquaculture

So far, very little attention has been paid to bacteria in aquaculture and their presence has normally been found to be associated with the control of bacterial diseases. The interaction between bacteria and microalgae involves different mechanisms, including growth stimulatory or inhibitory compound production, cross-signalling, and, generally speaking, the natural capacity of microalgae to adhere to associated specific microorganisms [56]. The selection of the appropriate microorganism consortia might greatly enhance the productivity, efficiency and sustainability of aquaculture [79]. In this sense, bacteria can stimulate algal growth, which is the key component of diet in aquaculture of other organisms. Therefore, a healthy feed supply would comprise grazers, algae and their associated beneficial bacteria [106]. The co-ingestion of algae and bacteria, for example, has been reported to result in healthier *Artemia* cultures, possibly through better nitrogen assimilation [107]. Moreover, the potential of microalgae in controlling pathogenic bacteria in aquaculture systems has been established at the laboratory experimental scale [59]. Other studies have dealt with algal-bacterial treatment of aquaculture wastewater with the use of resulting harvested biomass as feed for Pacific white shrimps, *Litopenaeus vannamei*, in the context of integrated, sustainable and recyclable aquaculture systems [79,108]. In addition, well-selected consortia of microalgae and bacteria might also lead to a better shellfish larval settlement [79]. The current lack of knowledge leads to several key challenges. In this context, it is important to get a deeper insight of the specific bacterial species naturally associated to algae. This involves, in relation to aquaculture, the diversity of the bacteria-microalgae interaction mechanisms and the understanding of the chemistry involved.

## 5. Conclusions

Microalgae-bacteria interactions are complex. At present, fragmentary knowledge has already been gathered on the chemical nature of a number of exchanged mediator molecules, including nutrients, which clearly regulate the relationship between microalgae and bacteria. Amino acids and vitamins have been identified among the main mediator molecules that regulate the relationship between microalgae and bacteria. The suitable control of such chemical interaction has been proposed as an efficient tool to increase yields and reduce costs in microalgae cultivation. Nevertheless, limited information at the molecular level is still available. It must be emphasized that science is just starting to reveal a minor fraction of the knowledge required to understand the chemistry behind the interactions of microalgae and bacteria. The chemical complexity of the microalgae-bacteria interactions includes a wide variety of molecular signals, exchanged metabolites, transporters and the molecules whose functions still have to be investigated. A greater insight at molecular level in the regulation of microalgae-bacteria interactions with sequenced organisms will enable the driving of specific algal-bacterial systems to produce the desired effects. The current development in the understanding of these interactions is leading to specific biotechnological applications, for instance, in the fields of wastewater treatment, bioremediation and sustainable aquaculture. Emerging technologies derived from microalgae-bacteria interactions are being developed in the field of energy generation. Among them, phototrophic microbial fuel cells are worth mentioning, although they still need development to be commercialized. In a world with an exponentially increasing demand of microalgal biomass for novel applications, one of the key challenges would definitely be the controlled integration of specific bacteria in the massive production processes of a specific microalga. Such integration should be aimed at the cost reduction of nutrients, algal biomass harvesting and intracellular algal product recovery. The overall process should hopefully become more sustainable by means of reducing the use of synthetic chemicals and the energy demand.

## Figures and Tables

**Table 1:** Examples of microalgae-bacteria interactions having positive effects on algal growth and accumulation of valuable compounds.

Microalga	Bacterium	Mediators from Microalgae	Mediators from Bacteria	Reference
Algal growth improvement/production cost decrease				
*E. huxleyi*	*P. gallaeciensis*	Dimethylsulphonio-propionate	Promoters and antibiotics	Seyedsayamdost *et al.* (2011) [53]
*B. braunii*	*Rhizobium* sp.		AHL	Rivas *et al.* (2010) [56]
*L. rostrate*	*M. loti*		Vitamin B_12_	Kazamia *et al.* (2012) [16]
*T. pseudonana* CCMP1335	*R. pomeroyi* DSS-3	2,3-dihydroxy-propane-1-sulfonate	Vitamin B_12_	Durham *et al.* (2015) [41]
*S. trochoidea*	*Marinobacter*	Organic molecules	Vibrioferrin	Amin *et al.* (2009) [22]
*S. trochoidea*	*Roseobacter*	Organic molecules	Vibrioferrin	Amin *et al.* (2009) [22]
*N. oleoabundans*	*A. vinelandii*		Siderophore	Santos *et al.* (2014) [48]
*Scenedesmus sp.*	*A. vinelandii*		Siderophore	Santos *et al.* (2014) [48]
Accumulation of fatty acids and lipids				
*C. vulgaris*	*A. brasilense*		Siderophore mediated nitrogen fixation	Leyva *et al.* (2014) [50]
Heterotrophic accumulation of starch and carbohydrates				
*C. vulgaris*	*A. brasilense*		Siderophore mediated nitrogen fixation	Choix *et al.* (2012) [51]
*C. sorokiniana*	*A. brasilense*		Siderophore mediated nitrogen fixation	Choix *et al* (2012) [51]
Photoautotrophic accumulation of starch and carbohydrates				
*C. vulgaris*	*A. brasilense*		Siderophore mediated nitrogen fixation	Choix *et al* (2012) [52]
*C. sorokiniana*	*A. brasilense*		Siderophore mediated nitrogen fixation	Choix *et al* (2012) [52]
